# Topical Application of Imiquimod as a Treatment for Chromoblastomycosis

**DOI:** 10.1093/cid/ciu168

**Published:** 2014-03-14

**Authors:** Maria da Glória Teixeira de Sousa, Walter Belda, Ricardo Spina, Priscila Ramos Lota, Neusa Sakai Valente, Gordon D. Brown, Paulo Ricardo Criado, Gil Benard

**Affiliations:** 1 Laboratory of Medical Investigation Unit 53, Division of Clinical Dermatology; 2 Laboratory of Medical Mycology, Tropical Medicine Institute; 3 Division of Clinical Dermatology, Clinics Hospital, Medical School, University of Sao Paulo, Brazil; 4 Aberdeen Fungal Group, Institute of Medical Sciences, University of Aberdeen, United Kingdom

**Keywords:** Imiquimod, chromoblastomycosis, *Fonsecaea pedrosoi*, innate immunity, antifungal treatment

## Abstract

Chromoblastomycosis is a subcutaneous mycosis that remains a therapeutic challenge, with no standard treatment and high rates of relapse. On the basis of our recent discoveries in mouse models, we tested the efficacy of topical applications of imiquimod to treat patients afflicted with this chronic fungal infection. We report results of treatment for the first 4 recipients of topical imiquimod, all of whom displayed a marked improvement of their lesions, both with and without concurrent oral antifungal therapy.

Chromoblastomycosis (CBM) is a chronic subcutaneous mycosis endemic in some tropical and subtropical areas that is caused by a group of dematiaceous fungi [1]. *Fonsecaea pedrosoi* is the most frequent etiological agent. Patients are infected through the inoculation of hyphae or conidia following transcutaneous trauma. Clinically, the lesions evolve progressively with erythema, papules, nodules, verrucous plaques, and/or ulcerations. Areas mostly affected are the lower limbs, followed by the upper limbs and, less commonly, the buttocks, trunk, and face. CBM is difficult to treat and has a low cure rate [2]. Many therapeutic approaches have been reported, including intravenous (amphotericin B) or oral (ie, 5-fluorocytosine, itraconazole, and terbinafine) antifungals, surgical excision, and physical treatments (ie, cryotherapy and thermotherapy), used alone or in combination. However, there is currently no gold standard therapy for CBM. The few treatment trials available report widely variable success rates, with some studies reporting relapses in up to 80% of patients [2].

We recently explored the mechanisms underlying susceptibility to infection with *F. pedrosoi*, using mouse models [3]. We discovered an underlying defect in innate recognition of this organism by Toll-like receptors (TLRs) that could be restored by exogenous administration of TLR agonists, including imiquimod. Remarkably, such treatment resolved the infection in mice without adverse effects [3].

Imiquimod, an imidazoquinoline, is a synthetic compound with potent antiviral, antitumor, and immunoregulatory properties that stimulates both the innate and acquired immune pathways through activation of TLR7. Imiquimod (Aldara; 3M Pharmaceuticals) was approved in 1997 by the US Food and Drug Administration for topical treatment of external anogenital warts, actinic keratosis, and superficial basal cell carcinoma [4]. Besides these approved indications, there have been sporadic case reports and studies suggesting its effectiveness in the treatment of other infectious and noninfectious cutaneous diseases, including cutaneous leishmaniasis [5]. Here, we present evidence that topical administration of imiquimod has a beneficial effect in the management of CBM.

## CASE REPORTS

The clinical features, diagnosis, histopathological findings, treatments, and clinical courses for all patients are summarized in Table 1.

**Table 1.** Summary of Data From the Patients With Chromoblastomycosis Treated With Imiquimod Alone or in Combination With Oral Antifungals

Variable	Case 1	Case 2	Case 3	Case 4
Clinical features	Erythematous infiltrated and verrucous lesion with crusts on right forearm for 2 y	Erythematous and infiltrated lesion with a keratotic and verrucous elevated surface with black dots on the dorsum of right hand for 3 y	Erythematous and infiltrated verrucous plaque lesion with black dots on the surface of the dorsum of left hand for 6 y	Erythematous and infiltrated lesion with verrucous surface on the right wrist for 5 y
Diagnosis	*F. pedrosoi* on culture of smear of the lesion	*F. pedrosoi* on culture of smear of the lesion	*F. pedrosoi* on culture of smear of the lesion	*F. pedrosoi* on culture of smear of the lesion
Histopathological findings on admission	Epidermis: hyperkeratosis with acanthosis, spongiosis, and microabscesses; dermis: lymphohistiocytic inflammatory infiltrate with plasma cells, Langerhans-type giant cells, and granulomas containing sclerotic cells	Hyperkeratosis, vacuolar degeneration of the basal layer, clusters of Langerhans cells, and giant cells containing sclerotic cells	Epidermal acanthosis and hyperkeratosis in a pseudo-epitheliomatous pattern; dermis: chronic inflammatory infiltrate with high numbers of neutrophils and several sclerotic cells	Epidermis: hyperkeratosis, acanthosis, vacuolar degeneration of the basal layer, and microabscesses; dermis: granulomatous infiltrate with giant cells containing sclerotic cells
Prior treatment (duration in mo)	…	ITRA (7)	ITRA + TERB (12)	…
Treatment (duration in mo)	IMQ + ITRA (17)	IMQ (6)	IMQ + ITRA + TERB (6)	IMQ (6)
Inflammatory exacerbation	Diagnosed at week 2 after treatment; biopsy showed lichenoid infiltration	Diagnosed at week 4 after treatment	Persisted up to 4 mo after treatment initiation	Diagnosed at week 4 after treatment; biopsy showed lichenoid infiltration
Clinical course	Cure after 20 mo of posttreatment follow-up	Clinical improvement with healed aspect but still positive for fungi; oral antifungal treatment (ITRA + TERB) was associated	Partial improvement; still receiving treatment	Healed aspect except for a small area still positive for fungi; oral antifungals (ITRA + TERB) administered for an additional 9 mo resulted in complete healing, with negative results of tests for fungi

Abbreviations: *F. pedrosoi*, *Fonsecaea pedrosoi*; IMQ, topical imiquimod 5%; ITRA, itraconazole; TERB, terbinafine.

### Case 1

**Figure 1.**
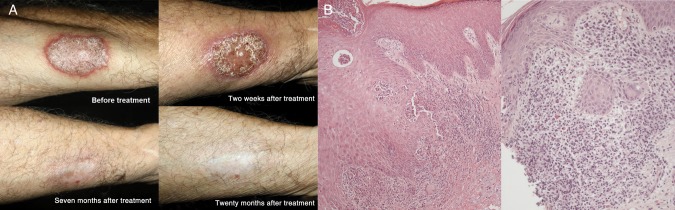
*A*, Pictures of the lesion on the forearm of case 1 before and during treatment with topical imiquimod 5% plus itraconazole 200 mg/day, as indicated. *B*, Findings of hematoxylin-eosin staining of the chromoblastomycosis lesion from case 1 before (left; original magnification × 100) and during (right; original magnification × 200) treatment with topical imiquimod and itraconazole.

Case 1 was a 71-year-old man with a 2-year history of a lesion on the right forearm (Figure 1*A*). Culture of a smear of the lesion yielded *F. pedrosoi*. Topical imiquimod 5% (5 times/week) and itraconazole (200 mg/day) were prescribed. After 2 weeks, the inflammatory aspect of the lesion was exacerbated, with a more infiltrated and verrucous surface and more enlarged and erythematous margins (Figure 1). Histopathological analysis revealed a lichenoid lymphocytic infiltrate in the dermis (Figure 1*B*). No sclerotic bodies or other fungal structures were identified in this biopsy section. After 7 months of the combination treatment, the exacerbated inflammatory process had subsided, leaving a healing aspect (Figure 1). After 10 months of treatment, the lesion further improved, presenting a fully healed aspect. Both treatments were then halted, and by 20 months after treatment only a superficial scar could be seen (Figure 1). Findings of direct mycological examination was negative.

### Case 2

Case 2 was a 70-year-old man with a 3-year history of a progressive lesion on the dorsum of the right hand (Supplementary Figure 1). Mycological examination of the lesion revealed sclerotic cells, which, on culture, yielded *F. pedrosoi*. The patient had been treated with itraconazol (400 mg/day) for 7 months, with no improvement. Treatment with topical imiquimod 5% 5 times weekly was started. Four weeks later, there was enhancement in the verrucous and infiltrative aspect of the lesion, as observed in the first patient (Supplementary Figure 1). This aspect was transitory, and by 14 weeks there was resolution of this inflammatory response and marked improvement of the lesion (Supplementary Figure 1). By 6 months, the lesion was apparently healed (Supplementary Figure 1). However, direct mycological examination of material obtained by vigorously scraping the lesion still yielded fungi. These organisms differed from the typical sclerotic bodies (with yeast-like form and binary division) seen before treatment and presented as elongated filamentous forms (Supplementary Figure 2). Because of the persistent presence of fungi, oral antifungals (itraconazole 400 mg and terbinafine 250 mg daily) were then administered along with continued topical application of imiquimod. At the last visit, after 3 months of the combination treatment, the patient presented with only an erythematous cicatricial macula, but the mycological examination was still positive.

### Case 3

Case 3 was a 46-year-old man with a 6-year history of a plaque lesion on the left hand (Supplementary Figure 3). Sclerotic cells were identified on direct mycological examination and by histopathological analysis (not shown). The patient was treated with itraconazole (400 mg) plus terbinafine (250 mg) daily for 1 year, with no improvement. Topical application of imiquimod 5% 5 times weekly was introduced in association with the orally administered drugs. As in the previous cases, treatment with imiquimod resulted in increases in the erythematous and verrucous aspects of the lesion that persisted up to 4 months of the combination treatment, after which the lesion gradually decreased in size (Supplementary Figure 3). Direct mycological examination of the lesion showed atypical filamentous sclerotic bodies, as observed in case 2. By 6 months, the lesion showed partial improvement, with reductions in the inflammatory process and the number of black dots (Supplementary Figure 3).

### Case 4

Case 4 was a 71-year-old man with a 5-year history of a lesion on the right wrist (Supplementary Figure 4). Mycological examination and histopathological analysis of the lesion revealed sclerotic cells. The patient was treated with topical imiquimod 5% cream as a monotherapy 4 times weekly. Four weeks later, there was an exacerbation of the inflammatory and verrucous aspect of the lesion (Supplementary Figure 4) that, on histopathological analysis, coincided with the presence of a lichenoid lymphocytic infiltrate in the dermis, which disrupted the dermal-epidermal junction in some places (Supplementary Figure 5). Thereafter this inflammatory process gradually subsided, and by 6 months the lesion was healed except for a small area located at the medial border, which still presented a slightly verrucous aspect (Supplementary Figure 4). Direct mycological examination of this area showed few fungal cells with elongated filamentous forms. We then prescribed itraconazole (200 mg) plus terbinafine (250 mg) daily in addition to the topical treatment with imiquimod. At the last visit, 9 months after initiation of the combination treatment, the lesion was in most part cicatricial, and mycological examination of the small verrucous area had negative findings.

## DISCUSSION

Here we have reported findings about the applicability of using topical imiquimod as a treatment for CBM. The first case demonstrated that imiquimod could modify and accelerate the response to conventional antifungal treatments. This encouraged us to test imiquimod in the treatment of patients in whom antifungal drugs yielded poor responses. In both patients in whom oral antifungals (itraconazole plus terbinafine) failed to induce improvement of the lesions (cases 2 and 3), the initiation of topical imiquimod treatment yielded marked improvement. In the fourth patient, imiquimod therapy alone resulted in a sharp clinical improvement but was apparently not able to completely eradicate the fungus. Of note, none of our patients reported any of the side effects occasionally related to topical imiquimod use, such as itching and a burning sensation [6].

Two interesting observations emerged from this study. In cases 2, 3, and 4, direct mycological examination of the lesion during imiquimod treatment showed that the sclerotic cells changed their morphology from the typical yeast form with binary division, normally seen in lesions, to an elongated filamentous form that has not been previously reported. This change in morphology is likely to represent a fungal response to the new pattern of local immune reactivity induced by imiquimod. In all patients we observed a transitory exacerbation of the verrucous and infiltrative characteristics of the lesion that preceded its gradual evolution to healing. In the patients who had a concomitant histopathological reevaluation, the inflammatory exacerbation coincided with a high influx of lymphocytes and histiocytes, characterizing a lichenoid reaction. This infiltrate denotes a change in the pattern of the inflammatory response toward an augmented cellular immunity and may be related to the previously described property of imiquimod of inducing psoriasis-like skin inflammation [7]. This inflammation was characterized by epidermal hyperproliferation, abnormal differentiation, epidermal accumulation of neutrophils in microabscesses, neoangiogenesis, and infiltrates consisting of CD4^+^ T cells, CD11c^+^ dendritic cells, and plasmacytoid dendritic cells.

Imiquimod is an immune response modifier that increases local cytokine production, with a subsequent activation of both the innate and adaptive immune systems [8]. It has been shown that patients with CBM may present T-helper type 1 hyporesponsiveness to *F. pedrosoi* antigens [9]. This deficiency was only partial and transitorily restored with conventional treatments [10], an observation that may help explain the high rate of relapses. Innate immunity mechanisms apparently fail to control the infection and to instruct appropriate adaptive immune responses, probably contributing to the chronic nature of the infection [3]. Activated macrophages are unable to kill the conidia, although they may present some fungistatic activity [11]. Thus, as we found in our animal models [3], a defect in innate recognition results in a failure to mount robust inflammatory responses and causes susceptibility to infection. Topical application of imiquimod overcomes this defect, enhancing inflammatory responses that lead to clearance of the pathogen and resolution of the infection. Such defects could also underlie susceptibility to other subcutaneous mycoses like eumycetoma, sporotrycosis, hyalohyphomycosis, and as such these mycoses may also benefit from administration of topical imiquimod [12].

Further studies using larger number of patients are required to confirm the beneficial effect of imiquimod observed here. These studies should also address the important issues of optimal dosage and treatment duration. Nevertheless, despite the limitations of a study involving a few patients, we believe that our observations strongly suggest the use of this topical immunomodulator as an adjuvant in the therapy of CBM.

## Supplementary Data

Supplementary materials are available at *Clinical Infectious Diseases* online (http://cid.oxfordjournals.org). Supplementary materials consist of data provided by the author that are published to benefit the reader. The posted materials are not copyedited. The contents of all supplementary data are the sole responsibility of the authors. Questions or messages regarding errors should be addressed to the author.

Supplementary Data
