# Supplementary material for: Topical Application of Imiquimod as a Treatment for Chromoblastomycosis
Source: Clin Infect Dis. 2014 Mar 14;58(12):1734–7. doi: 10.1093/cid/ciu168 (PMC4036686; doi:10.1093/cid/ciu168)
Supplement: Supplementary Data [file supp_58_12_1734__index.html]

Topical Application of Imiquimod as a Treatment for Chromoblastomycosis — Topical Application of Imiquimod as a Treatment for Chromoblastomycosis — Supplementary Data 

# Topical Application of Imiquimod as a Treatment for Chromoblastomycosis

## Supplementary Data

Supplementary Data

**Files in this Data Supplement:**

- Supplementary Data - Doc file
