# Supplementary material for: Topical Application of Imiquimod as a Treatment for Chromoblastomycosis
Source: Clin Infect Dis. 2014 Mar 14;58(12):1734–7. doi: 10.1093/cid/ciu168 (PMC4036686; doi:10.1093/cid/ciu168)

Supplementary Figure 1: Pictures of the lesion on the dorsum of the hand of patient 2 before and during treatment with topical imiquimod 5%, as indicated.

Supplementary Figure 2: Sclerotic bodies with altered morphology (elongated filamentous form) of the lesion of patient 2 after treatment with imiquimod as compared with before treatment (insert).

Supplementary Figure 3: Pictures of the lesion on the hand of patient 3 before and during treatment with topical imiquimod 5% plus itraconazole and terbinafine, as indicated.

Supplementary Figure 4: Pictures of the lesion on the wrist of patient 4 before and during treatment with topical imiquimod 5%,, as indicated.

Supplementary Figure 5: Histopathology of the chromoblastomycosis lesion of patient 4 before (left) and during topical imiquimod monotherapy (right) (HE OM x100).


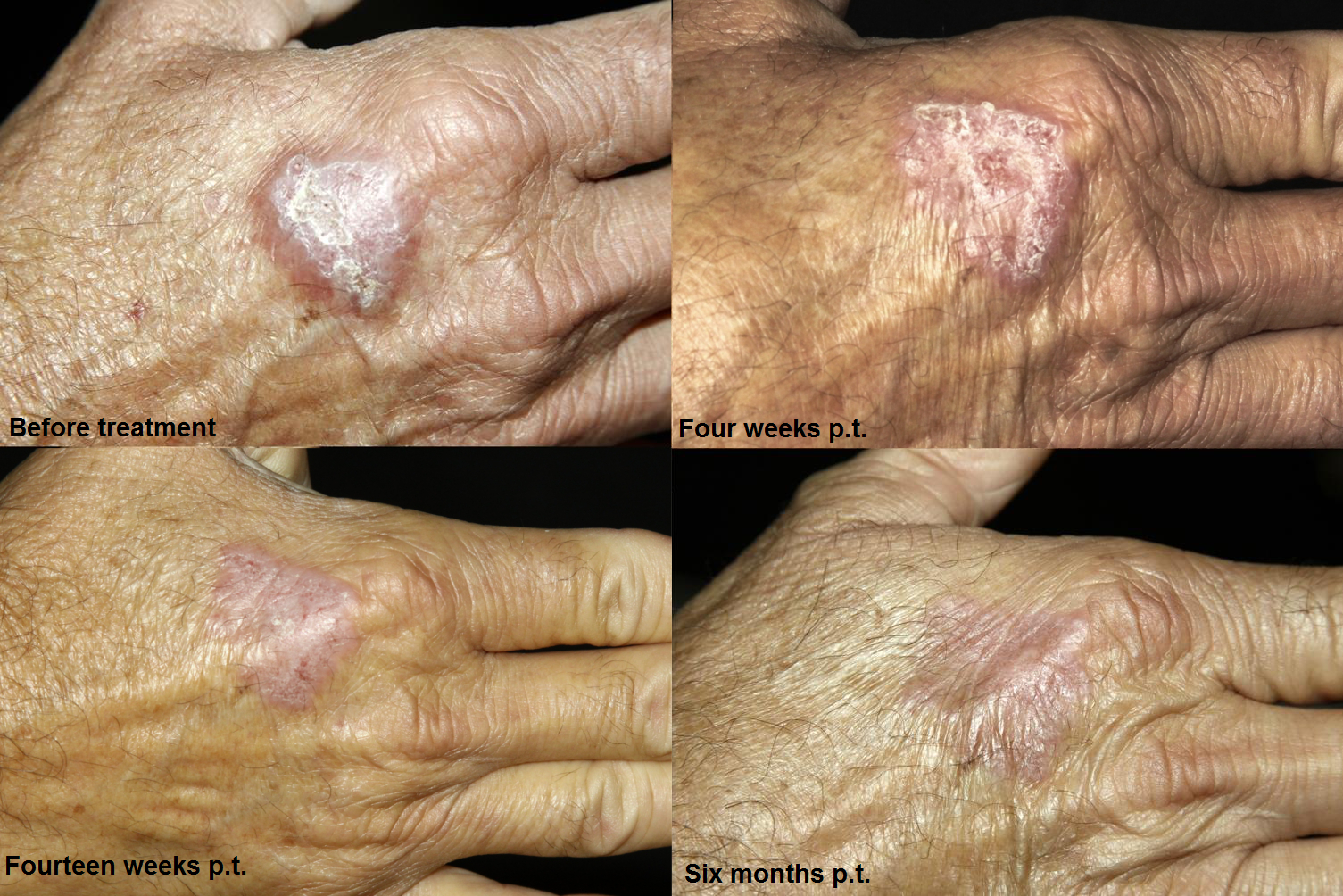


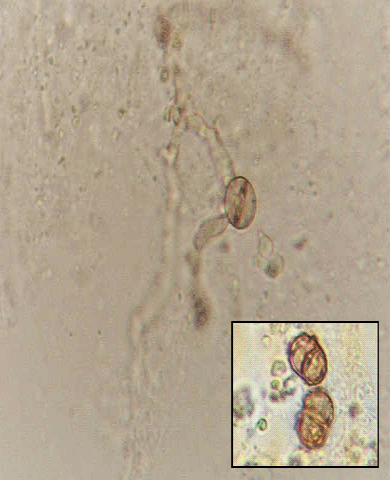


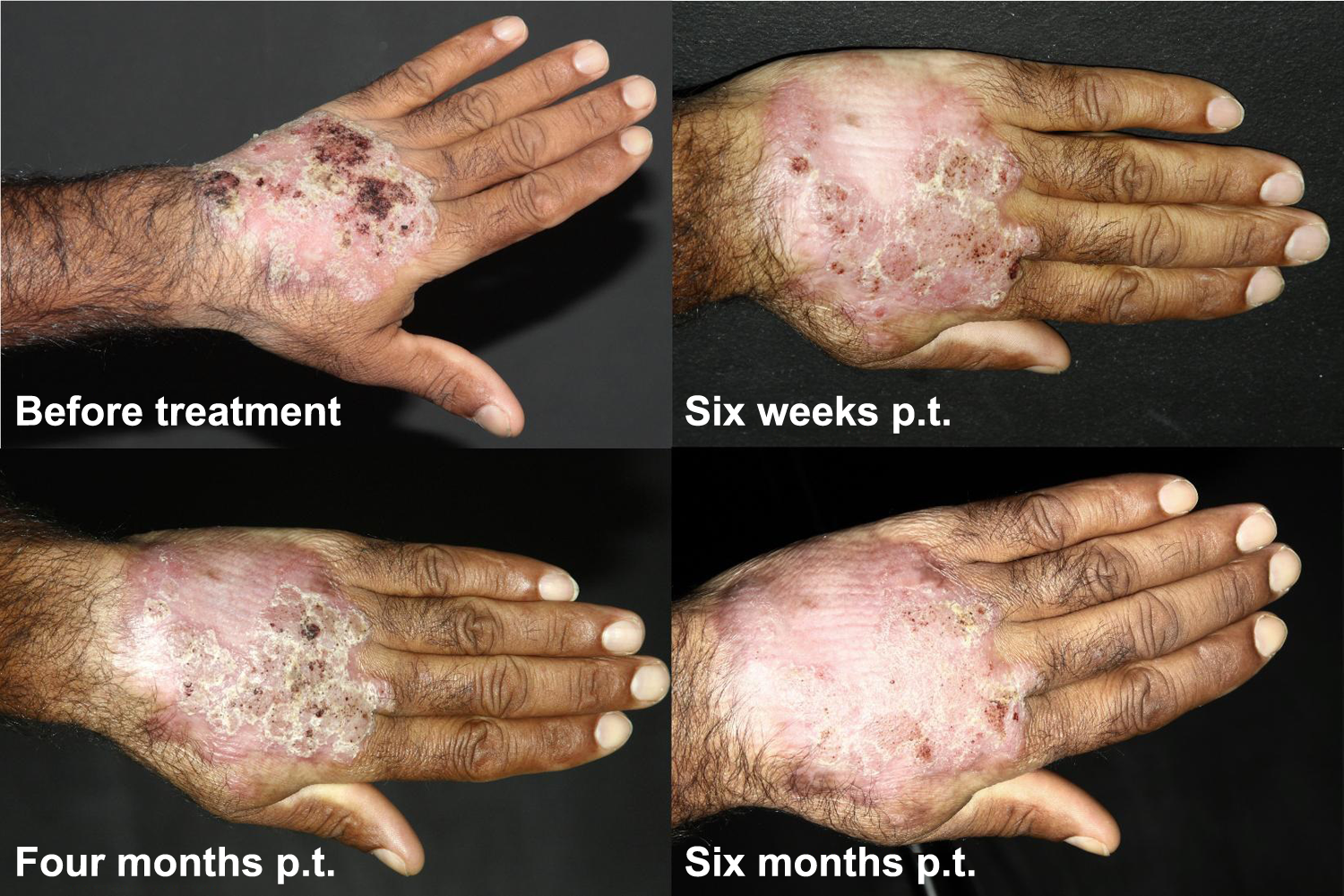


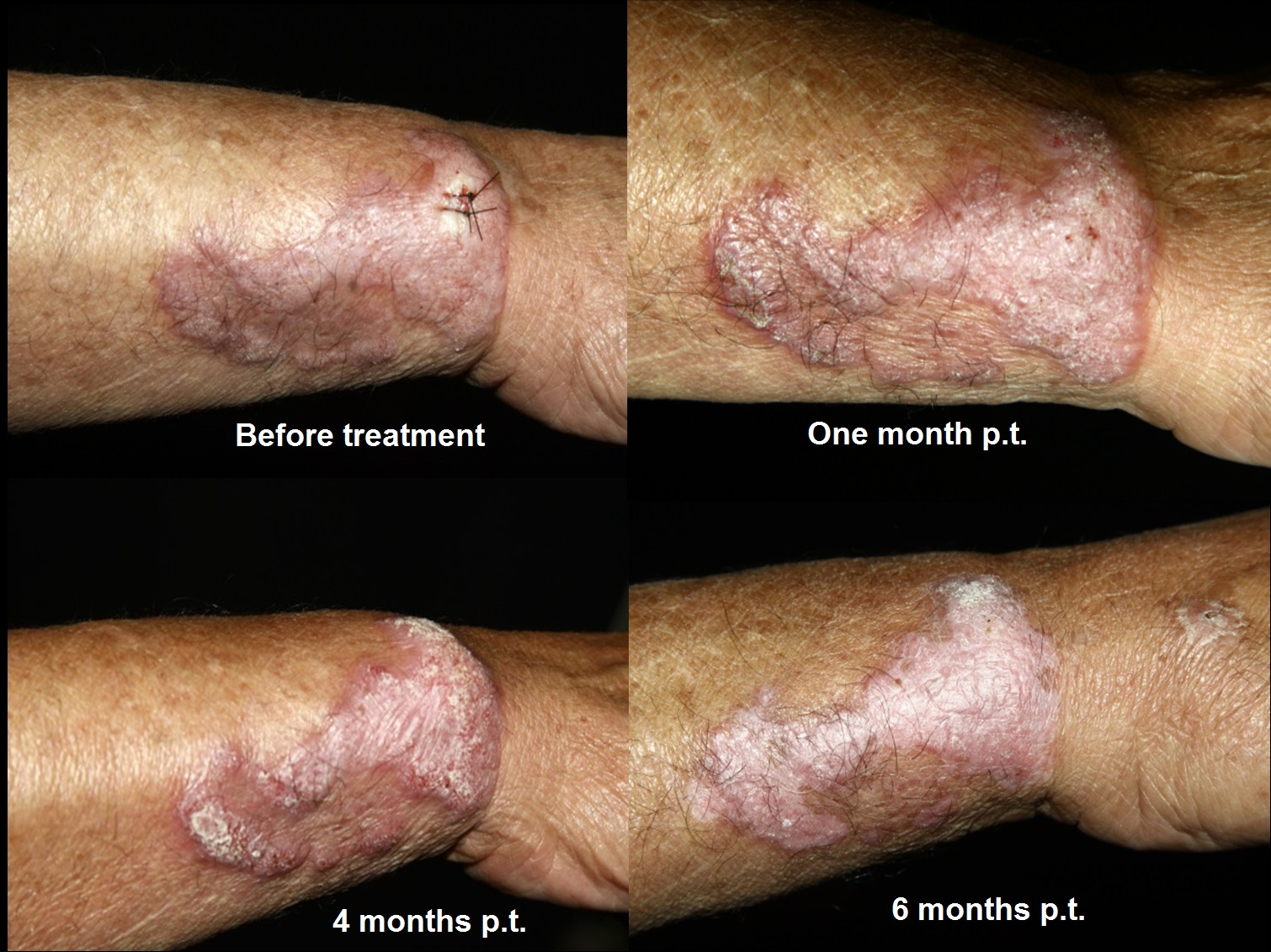


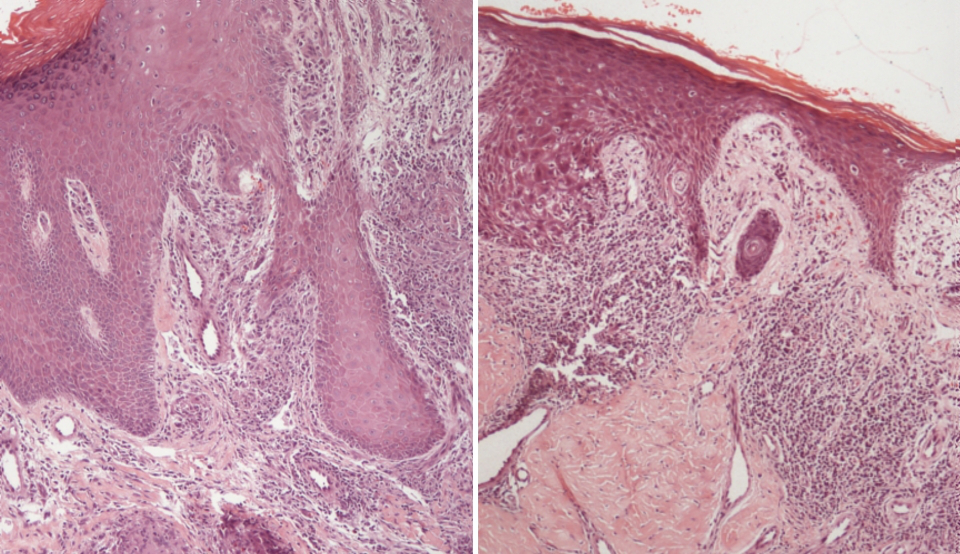

Supplement: Supplementary Data [file supp_ciu168_ciu168supp.doc]
